# Nursing in Prisons

**Published:** 1898-06-11

**Authors:** 


### Editor's Letter-Box.

[Our Correspondents are reminded that prolixity is a great bar to publication, and that brevity of style and conciseness of statement greatly facilitate early insertion.]

### NURSING IN PRISONS.

Miss Honnor Morten writes: On my way to Wormwood Scrubs to lecture in the prison there yesterday I bought a copy of The Hospital, and was astonished at the way in which the article on page 174 contradicted the more recent news *re* prison nursing you gave two weeks ago in "The Mirror." The facts are according to the paragraph which stated that the Home Secretary had arranged for re-organisation of the hospital staff in big prisons so that trained persons should have care of the sick, and that smaller prisons are instructed to place themselves in communication with local nursing institutions and get help when necessary. As a matter of fact, the infirmary matron at Wormwood Scrubs was engaged in institution nursing three years before she went to the prisons. It may be that prison infirmaries are in some places behind the times—I have not the wide knowledge your writer claims—but as regards Wormwood Scrubs and Holloway I have visited both within the last fortnight and speak from personal experience.

